# Relationship Between Estimated Average Glucose (eAG) and Fasting Plasma Glucose in a Cohort of Pakistani Diabetic Subjects

**DOI:** 10.7759/cureus.18435

**Published:** 2021-10-02

**Authors:** Nanik Ram, Sibtain Ahmed, Saadia Sattar, Saira Furqan, Najmul Islam

**Affiliations:** 1 Endocrinology, Aga Khan University Hospital, Karachi, PAK; 2 Pathology & Laboratory Medicine, Aga Khan University Hospital, Karachi, PAK; 3 Medicine, Aga Khan University Hospital, Karachi, PAK

**Keywords:** pakistan, glycemic control, fasting, glucose, hba1c, estimated average glucose, diabetes mellitus

## Abstract

Introduction

Scientific literature is scarce on the utility of estimated average glucose (eAG) from Pakistan. Hence, there is a dire need to evaluate the relationship between eAG and fasting plasma glucose (FPG), in order to enhance our understanding of eAG and its usefulness. This study aims to investigate the relationship between FPG and eAG in diabetic patients calculated using HbA1C.

Materials and methods

A retrospective study was conducted at the Aga Khan University, Karachi, Pakistan. The medical records of both genders in the age range of 18-60 years, presenting as outpatients at the endocrine clinic, labeled as DM, were reviewed from January 2013 to December 2019. The subjects were divided into three groups A (<130 mg/dL), B (130-179 mg/dL), and C (>180 mg/dL) based on FPG levels. A correlation was compared using Spearman’s correlation. Box, whisker plots, and scatter plots were computed by R studio.

Results

After excluding those with missing values for either serum Cr, FPG, and HbA1c and sub-optimal renal function based on estimated glomerular filtration rate (eGFR) a total of 4,673 cases were analyzed. Subgroup A showed good glycemic control, whereas subgroup C showed poor glycemic control. The difference between eAG and FPG was significant (p < 0.0001). eAG values were found to be elevated than FPG values in groups A and B and almost the same in group C, whereas a moderately significant correlation with eAG and FPG in all three groups.

Conclusion

The correlation between eAG and FPG varies with blood glucose control and was significantly higher in the poorly controlled diabetes group. As the association between the FPG and eAG levels varies with the extent of blood glucose control, reporting eAG with HbA1c by a simple formula, at no additional cost will prove to be beneficial for clinical care.

## Introduction

Diabetes mellitus (DM) characterized by a defect of insulin secretion or insulin resistance is a chronic debilitating disorder [1]. In the long run, if DM goes uncontrolled, the metabolic complications and the persistent hyperglycemic state often leads to micro and macrovascular complications, affecting the overall quality of life [2]. The International Diabetes Federation has estimated a burden of 451 million people with DM globally [3]. As per the National Diabetes Survey of Pakistan (NDSP 2016-2017), the country-wide prevalence of diabetes is 26.3% and is expected to rise steeply, keeping in view the trend projected from the results of the previous surveys undertaken in the country [4].

Various longitudinal studies have suggested that adequate comprehension of the biochemical laboratory workup of DM leads to better outcomes and personalized glycemic targets [5]. Among the various biochemical markers associated with DM diagnosis and management glycated hemoglobin (HbA1c) is of utmost importance owing to its utility as a reliable marker to assess timely control over the preceding months [6]. Percent (%) of total hemoglobin or mmol/mol is the conventional approach for the expression of HbA1c values. Both expressions are not easily comprehensible for a DM patient with non-medical background [7]. 

To establish the utility of HbA1c as a marker of glycemic control various studies have evaluated its relationship with average glucose levels with conflicting results [8]. In 2008, Nathan et al. conducted the International HbA1c-derived average glucose (ADAG) trial, which established a linear dependence between HbA1c and averaged plasma glucose levels, and a simple mathematical equation for the calculation of estimated average glucose (eAG) level using the HbA1c level was introduced [9]. The relationship between A1C and eAG is described by the formula 28.7 x A1C - 46.7 = eAG.

This equation has been extensively evaluated since then, and citing eAG values with HbA1c laboratory reports has become a common practice in most developed countries [10]. Even though eAG has been the constant focus of most scientific guidelines, over many years since its inception, there is a scarcity of evidence from Pakistan to support or against the utility in the laboratories nationwide [11]. From a global perspective, data from the College of American Pathologists Survey shows that reporting eAG with lab has risen well over 50% [12].

However, most of the studies have been undertaken in Caucasians, and data from Pakistan are scarce. Furthermore, most clinical laboratories in Pakistan have not yet started reporting eAG values and a widespread understanding of its utility in the medical fraternity is missing.

HbA1c levels are also indirectly influenced by age, gender, race, and epigenetics. A study by Bergenstal et al. has revealed population differences in A1c levels, where it was significantly higher in blacks compared to white subjects, in relation to plasma glucose lower than white population [13]. Further evaluation of the eAG and HbA1c relationship becomes vital in the Pakistani population as the native southeast Asian population possesses a more potent diabetogenic phenotype and variable hemoglobin glycation rates compared to Caucasians [14].

With the high prevalence of DM in the country, the rising menace of obesity, and lack of health care facilities there is a need to evaluate the utility of eAG and its correlation with fasting plasma glucose (FPG) in a genetically heterogeneous Pakistani population. In the context of the dearth of literature and possible population-specific differences, this study was undertaken to further investigate this relationship in native Pakistani diabetic subjects.

## Materials and methods

A cross-sectional study was undertaken at the Aga Khan University, Karachi, Pakistan, in collaboration between the section of Endocrinology, Department of Internal Medicine, and section of Chemical Pathology, Department of Pathology, and Laboratory Medicine. The medical records of both genders in the age range of 18-60 years, presenting as outpatients at the endocrine clinic, labeled as DM type 2, were reviewed by a team consisting of a consultant chemical pathologist and endocrinologist from January 2013 to December 2019 after approval from the institutional ethical review committee (ERC #2020-5449-14410). The center is a tertiary care non-profit hospital, situated in the economic and financial hub, largest and the most densely populated city of Karachi, Pakistan. The hospital is the largest medical facility in Pakistan with many specialized sub-specialty medical services. It is accredited by the Joint Commission International (JCI) and the laboratory services are accredited by JCIA and College of American Pathologists (CAP) accredited. Being a national referral center, the population being served is representative of the entire geographical distribution of the country.

The biochemical investigations including FPG levels, HbA1c, and serum creatinine (Cr) levels undertaken on the same blood sample alongside the demographic details and the duration of DM were collected on a pre-structured questionnaire. The analysis was performed on the Adiva 1800 analyzer (Siemens Diagnostics) using the manufacturer's recommendations. The CKD-EPI Pakistan equation was used to calculate the estimated glomerular filtration rate (eGFR) [15]. Patients with an eGFR of below 60 mL/min/1.73 m^2^ were not included as compromised renal function is known to interfere with HbA1c analysis. eAG (in mg/dL) was computed using the equation “28.7 x HbA1c - 46.7” as validated previously and available from the American Diabetes Association (ADA) platform as a free online calculator.

FPG levels, i.e., Group A < 130 mg/dL, Group B 130-179 mg/dL, and Group C > 180 mg/dL were used to stratify the subjects group wise.

Histogram and Schapiro-Wilk test were performed for normality check. Mean and standard deviation was reported for continuous variables, whereas number and frequency were reported for categorical variable. A correlation was compared using Spearman’s correlation. Box, whisker plots, and scatter plots were computed by R studio, keeping a p-value of <0.05 as statistically significant.

## Results

**Figure 1:**
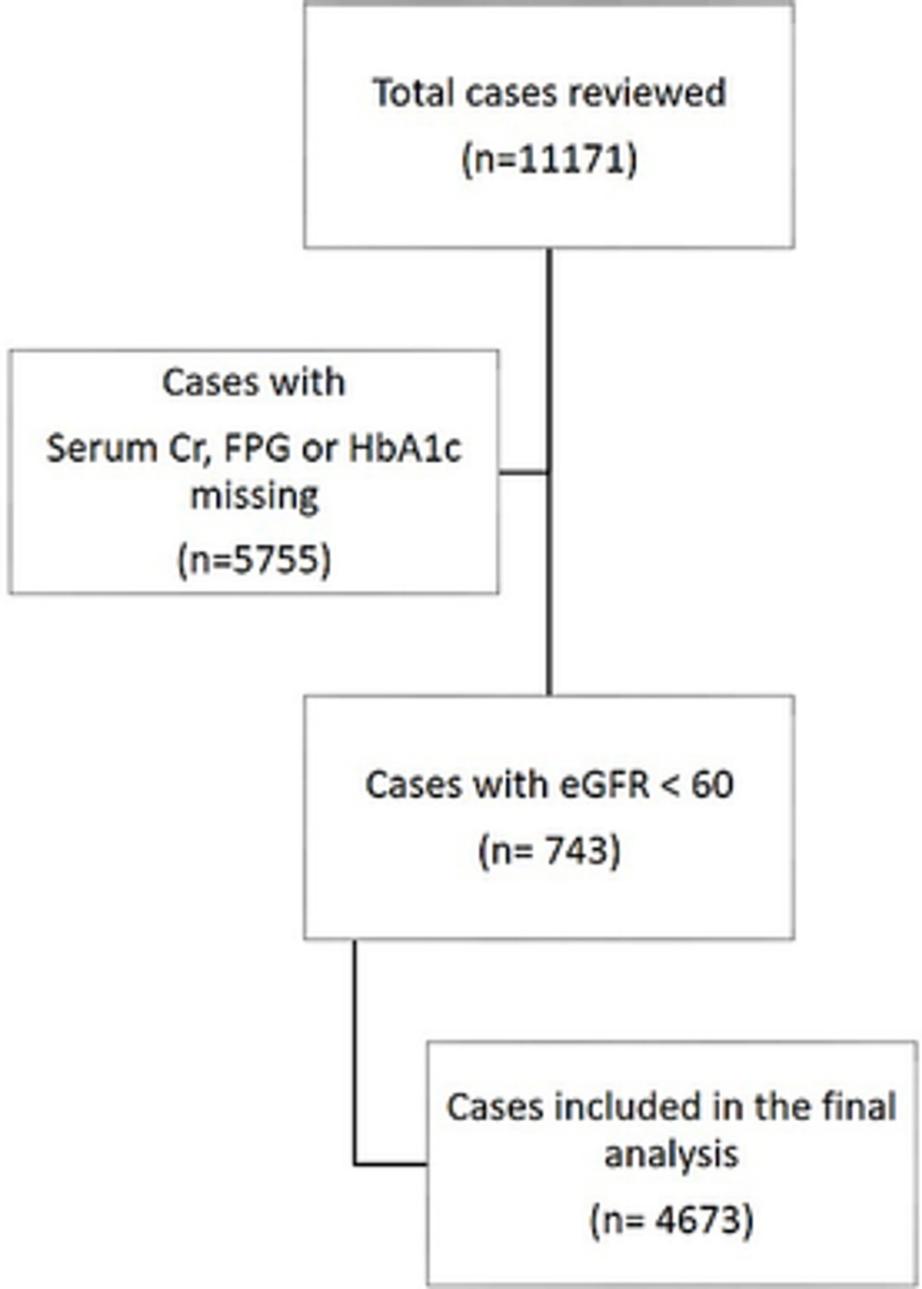
Flow diagram of data scrutiny of cases

A total of 11,171 cases were reviewed during the study duration. After excluding those with missing values for either serum Cr, FPG, and HbA1c and sub-optimal renal function based on eGFR a total of 4,673 cases were evaluated (refer to Figure 1).

**Figure 2:**
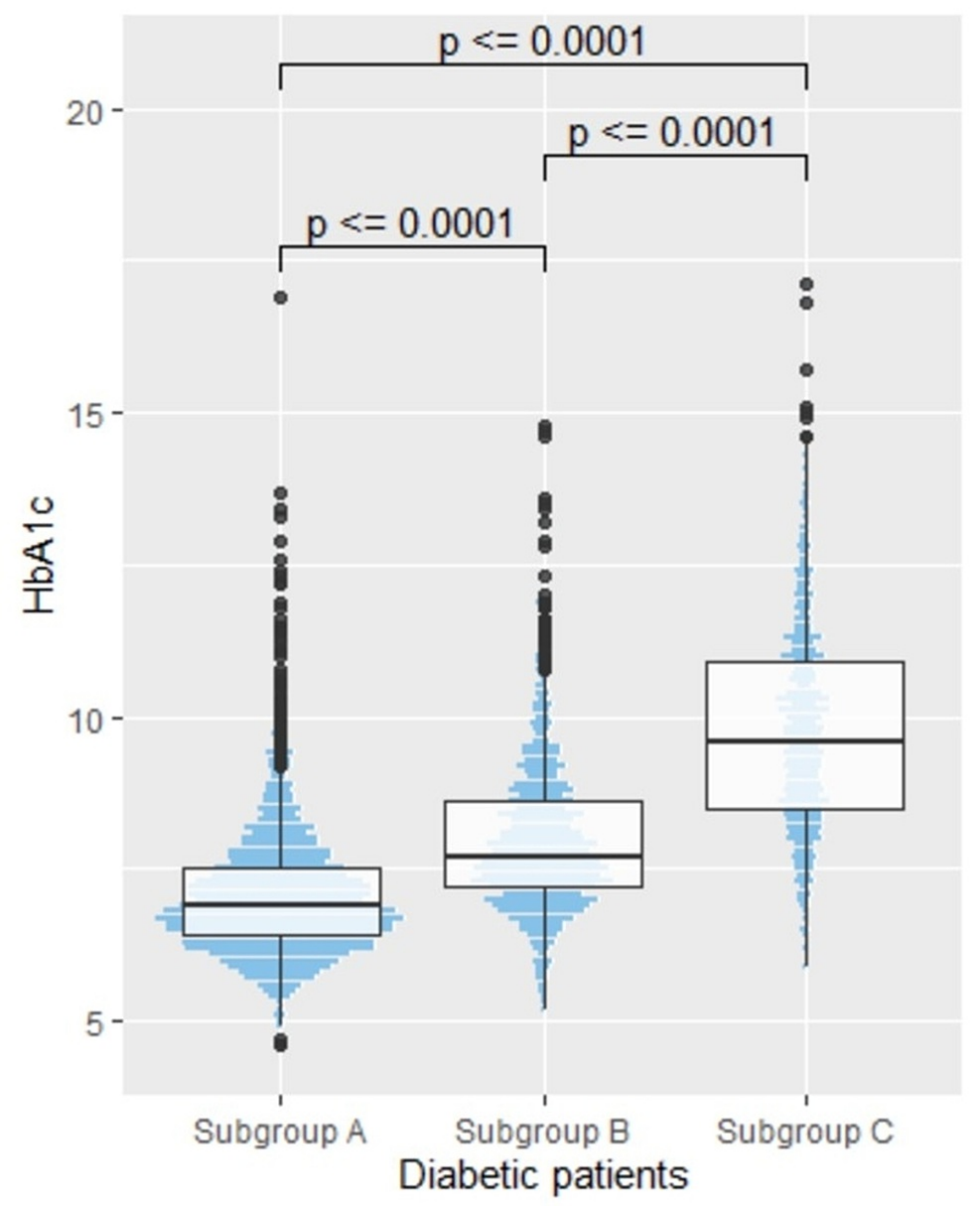
Combined dot plot and box-and-whisker plot of HbA1c levels in diabetic subjects (subgroups A, B, and C)

The subgroups A, B, and C were devised as taking values of 130 mg/dL and 180 mg/dL, based on ADA recommendations for pre- and post-prandial glucose controls [16]. Subgroup A revealed good control (HbA1c: 7.1 ± 1.1%), whereas subgroup C showed poor glycemic control (HbA1c: 9.8 ± 1.7%) (refer to Figure 2).

**Figure 3:**
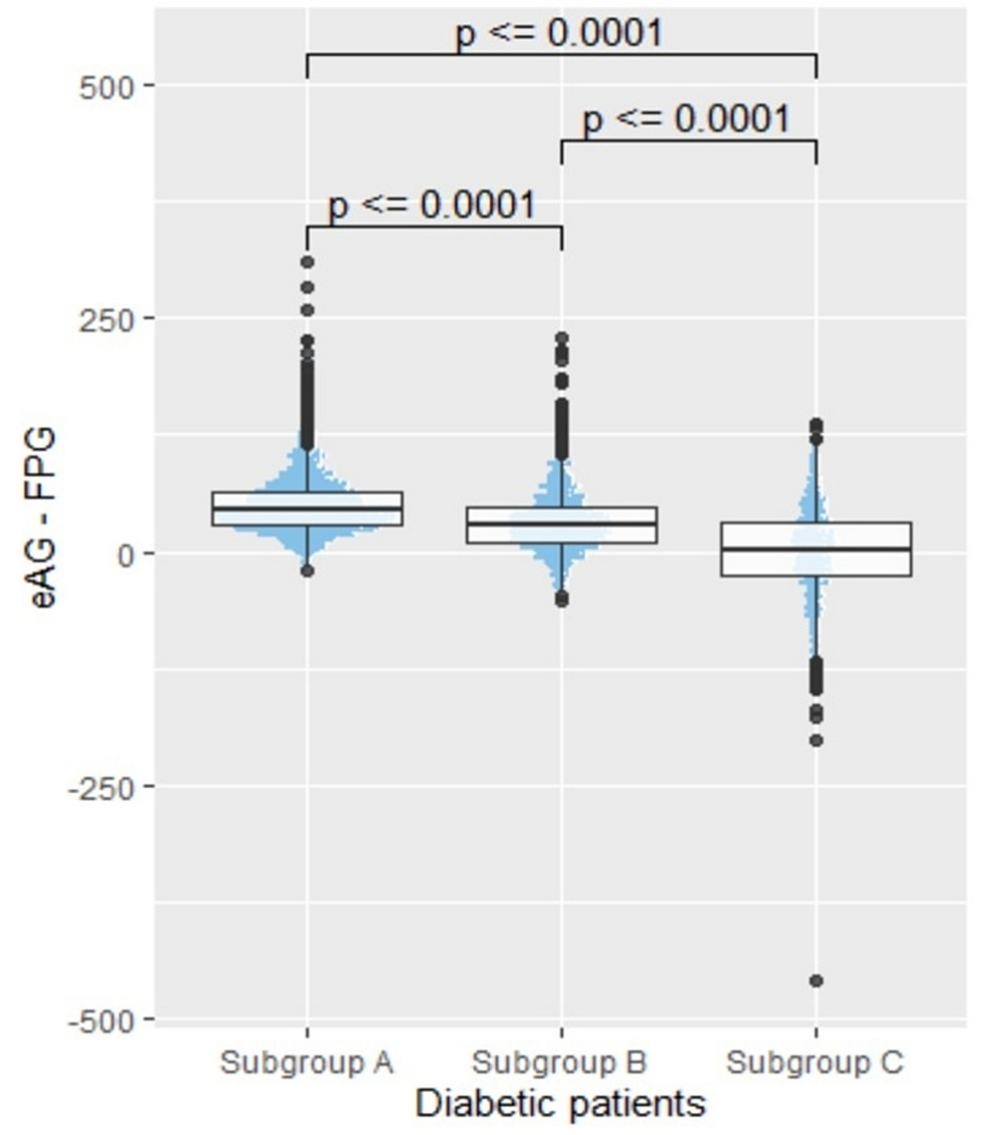
Combined dot plot and box-and-whisker plot of the difference, i.e., eAG-FPG eAG - estimated average glucose, FPG - fasting plasma glucose

Statistically significant (p < 0.0001) difference was noted between eAG and FPG values as depicted in Figure 3.

Except for subgroup C, eAG values were found to be higher than FPG values in groups A and B as depicted in Table 1.

**Table 1:** Characteristics of diabetic patients showing differences and correlation coefficients between eAG and FPG eGFR - estimated glomerular filtration rate, eAG - estimated average glucose, FPG - fasting plasma glucose

Subgroup FPG	A (<130 mg/dL)	B (130–179 mg/dL)	C (≥180 mg/dL)	Total
n	2,370	1,473	830	4,673
Age	55.7 ± 11.7	54.3 ± 11.3	51.2 ± 11.9	54.5 ± 11.7
Male:Female	1204:1166	776:697	406:424	2,386:2,287
Duration	9.2 ± 7.8	9.6 ± 7.4	10.2 ± 7.3	9.6 ± 7.6
Creatinine	0.8 ± 0.2	0.8 ± 0.2	0.8 ± 0.2	0.8 ± 0.2
eGFR	93.6 ± 16.6	94.3 ± 16.8	96.8 ± 18.2	94.4 ± 17.0
HbA1c	7.1 ± 1.1	8.0 ± 1.2	9.8 ± 1.7	7.8 ± 1.6
eAG	156.5 ± 31.3	181.9 ± 35.4	233.8 ± 47.8	178.2 ± 45.2
FPG	106.3 ± 14.9	148.9 ± 13.5	234.1 ± 52.3	142.3 ± 53.1
eAG – FPG	50.2 ± 32.4	32.9 ± 34.4	-0.4 ± 51.5	35.8 ± 41.4
Correlation between eAG and FPG	0.167	0.263	0.473	0.648

**Figure 4:**
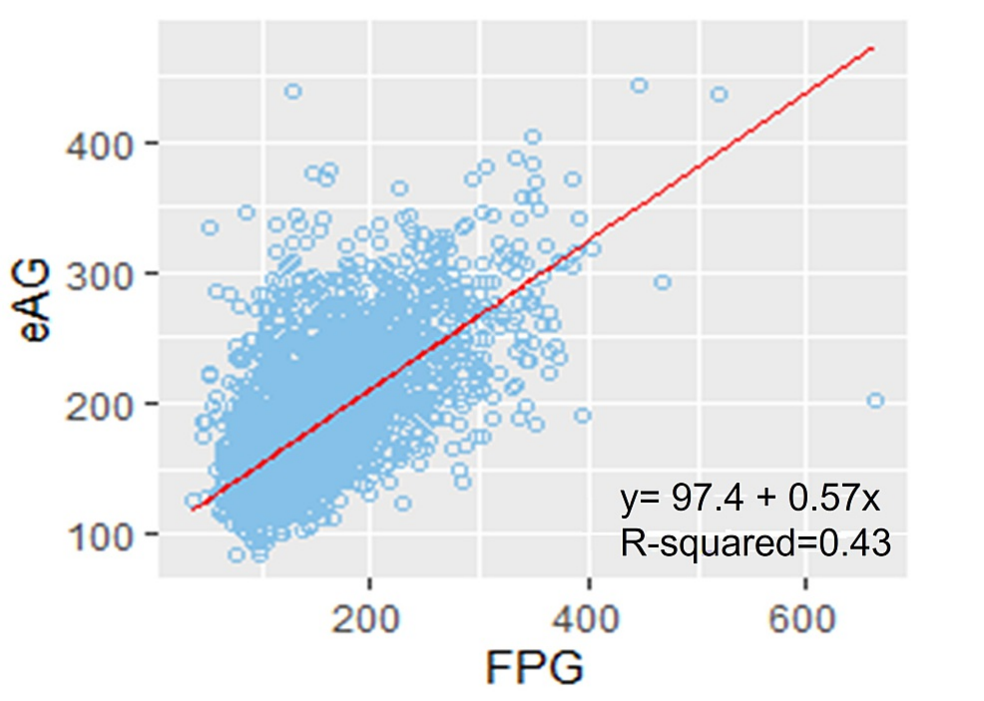
Relationship between FPG and eAG

FPG showed a reasonable significant correlation with eAG (r = 0.648, p < 0.0001) as shown in Figure 4 for all subjects, which showed a significant decline in stratification in subgroups as shown in Table 1.

## Discussion

Owing to their convenience and stabilization, FPG and HbA1c are popularly used in clinical practice for diabetics. The HbA1c has been widely adopted as a gold standard test for the evaluation of diabetic subjects in order to determine the long-term glycemic status [17]. One limitation often associated with HbA1c is the reporting units of mmol/mol and %, which differs from the usual units of blood glucose monitoring, i.e., mg/dL, often creating a confusing situation for the patients as well as clinicians for comprehension [18]. To overcome these limitations, international bodies including the ADA and the International Diabetes Federation proposed a mathematical expression termed eAG, which facilitates comprehension of HbA1c values in units parallel to self-monitoring [19]. Various guidelines recommend reporting eAG with every HbA1c report, however, it is not widely practiced by the majority of laboratories in Pakistan, and advocacy is required regarding its use based on evaluation in the local population [20]. With this perspective in mind, we planned to study the association between FPG and eAG in native Pakistani diabetic subjects.

This study demonstrated that corresponding to declining FPG levels, the differences between FPG and eAG demonstrated an increasing trend and the relationship significantly worsened. A similar trend was reported by Kim et al. in a cohort of 5,567 DM patients in Samsung Medical Center, Korea [21]. This phenomenon can be linked to postprandial glucose which also contributes to the determination of eAG levels alongside FPG [22]. Poor correlation was noted while comparing eAG with FPG in well-controlled DM, which better correlation was noted in subgroup B and C with poorly controlled status. Our results are in cohesion with Monnier et al. in type 2 diabetics [23]. The results show that no ethnic differences were noted for the native Pakistani population as evaluated by this study.

Moreover, the mean difference between eAG and FPG was negative in the poorly controlled subgroup C with 17% of the cases. Our results second Bozkaya et al, who reported similar observation in 3,891 diabetic patient samples in the Turkish population [18]. Another study by Rosediani et al in Malaysia diabetic subjects, established that postprandial plasma (PP) glucose and FPG correlated significantly with HbA1c but PP showed a better correlation with HbA1c than FPG (r= 0.604 vs. 0.575) [24]. This can be attributable to the morning hyperglycemia, i.e., the dawns phenomenon along with rising demand for insulin linked with a nocturnal growth hormone surge [25].

Furthermore, an interesting finding was noted that the majority of the cases had higher eAG levels than FPG levels. Whereas on evaluation of group C, contrasting results were revealed, i.e., eAG levels were lower than their FPG levels, as shown in Table 1. This can be linked to the assumption that subjects with higher FPG levels are often stressed before phlebotomy or non-compliant with anti-diabetic drugs since their FPG levels were higher than their average blood glucose levels.

Moreover, our findings depict that the relationship between HbA1c and FPG changed according to the different FPG ranges. When FPG was higher, the relationship was stronger in subgroup C. This is consistent with a Chinese study by Guan et al. that has reflected the variation with FPG values [26]. In a nutshell, reporting HbA1C value as eAG will be a “win win” situation for both patients and physicians, especially it will support to convince the patients of the importance of glycemic control.

There were few limitations of this study, notably, we did not have post-prandial and nocturnal glucose readings available to further study the relationship between eAG and FPG in depth. Moreover, as the complete blood picture was not available for all subjects anemic cases were not excluded. Nevertheless, no population base difference was noted as in most instances our results were in concordance with published studies from various other countries.

## Conclusions

In conclusion, eAG showed better performance in the poorly controlled diabetics with FBS > 180 mg/dL. For this vulnerable and care-sensitive group, eAG can serve as a swift and easily comprehensible measure way to indirectly determine average glucose levels with the same reporting units for self-blood glucose monitoring. This will supplement clinicians to facilitate care and counsel patients in a more intuitive way. Moreover, it can serve as a useful measure for clinical laboratories across Pakistan to enhance the quality of reporting at no added substantial cost. However, as the association between the FPG and eAG levels varies with the extent of blood glucose control, reporting eAG with HbA1c by a simple formula, at no additional cost will prove to be beneficial for clinical care.
